# Biofilm Formation Patterns of *S. epidermidis* (RP62A) and *S. aureus* (UAMS-1) Are Defined by Orthopaedic Implant Materials and Surface Wear

**DOI:** 10.3390/antibiotics15040338

**Published:** 2026-03-26

**Authors:** Tatyana Sevastyanova, Cornelia Loy, Barbara Schneider-Wald, Klaus Notarbartolo, Gregor Reisig, Stefanie Gaiser, Ali Darwich, Mohamad Bdeir, Alexander Blümke, Sascha Gravius, Andreas Schilder

**Affiliations:** 1 Department of Orthopaedic and Trauma Surgery, University Medical Centre Mannheim, Medical Faculty Mannheim, University of Heidelberg, Theodor-Kutzer-Ufer 1-3, 68167 Mannheim, Germany; th234@stud.uni-heidelberg.de (C.L.); barbara.schneider@medma.uni-heidelberg.de (B.S.-W.); gregor.reisig@medma.uni-heidelberg.de (G.R.); ali.darwich@umm.de (A.D.); alexander.bluemke@umm.de (A.B.); sascha.gravius@umm.de (S.G.); andreas.schilder@medma.uni-heidelberg.de (A.S.); 2 Endocon GmbH, In der Au 5, 69257 Wiesenbach, Germany; notarbartolo@endocon.de

**Keywords:** biofilm, staphylococcus epidermidis, staphylococcus aureus, orthopaedic implant materials, periprosthetic joint infection (PJI)

## Abstract

***Background/Objectives:*** *Staphylococcus epidermidis* (RP62A) and *Staphylococcus aureus* (UAMS-1) are clinically relevant pathogens frequently implicated in implant-associated infections due to their ability to form biofilms. RP62A is typically linked to persistent, chronic, low-grade infections, whereas UAMS-1 is associated with acute, invasive disease. Both strains serve as representative models for chronic and acute periprosthetic joint infections (PJIs). The objective of this study was to examine and compare in vitro biofilm formation by RP62A and UAMS-1 on orthopaedic materials/disc surfaces of defined composition. ***Methods:*** In vitro biofilm formation assays were performed using orthopaedic disc surfaces composed of cobalt–chromium alloy (CoCr), titanium alloy (Ti), and polyethylene (PE) after 72 h of incubation. Biofilm biomass was quantified using crystal violet staining, with absorbance measured at OD_570_. A polystyrene (PS) surface served as a control. Additionally, retrieved orthopaedic explant components were used as substrates for in vitro biofilm assays, in which RP62A was incubated for 72 h on the explanted surfaces. Supporting assays on glass slides were conducted to examine strain-specific biofilm-related architecture. ***Results:*** In vitro biofilm mass quantification assays showed strong biofilm formation by RP62A across all tested surfaces, with the highest absorbance on CoCr (OD_570_ = 5.80 ± 0.19). Notably, biofilm formation on CoCr was 76% higher compared to PS (*p* < 0.0001). No significant differences were observed among all three surface discs (*p* > 0.1). Biofilm formation was highest on PE for UAMS-1 (OD_570_ = 1.29 ± 0.09) and was significantly greater than on Ti (178%, *p* < 0.001) and CoCr (196%, *p* < 0.0001). In the in vitro assays performed on retrieved explant components, RP62A showed pronounced biofilm accumulation on polyethylene tibial inserts, particularly in regions of mechanical wear and friction. Supporting assays on glass slides were performed to examine strain-specific surface microstructural, revealing dense network-like structures for RP62A and thinner, discontinuous layers for UAMS-1. ***Conclusions:*** RP62A formed dense biofilms in vitro on multiple orthopaedic implant materials and retrieved explant components, consistent with its association with chronic periprosthetic joint infections. Increased biofilm accumulation was observed on mechanically worn polyethylene surfaces. In contrast, UAMS-1 showed lower biofilm formation on metallic disc surfaces, indicating strain- and material-dependent differences. These findings highlight the relevance of implant material selection and surface integrity for strategies targeting biofilm-associated implant infections.

## 1. Introduction

Implant-associated infections represent a critical clinical challenge due to their severity and resistance to treatment [1,2,3]. Current management strategies often necessitate prolonged antimicrobial therapy and surgical removal of the infected implant, leading to extended hospital stays, significant patient discomfort, and a high risk of reinfection in subsequent implantations [4,5,6,7,8]. Biofilm-forming pathogens pose a particular challenge to eradicate as biofilm formation is associated with increased infection severity and persistence [9,10,11]. The bacterial species *Staphylococcus aureus* and *Staphylococcus epidermidis* are clinically important biofilm-forming pathogens frequently implicated in implant-associated infections, including periprosthetic joint infections (PJIs). They form biofilms through initial adhesion to implant surfaces followed by extracellular matrix accumulation, producing structured bacterial communities with enhanced antimicrobial resistance—*S. epidermidis* biofilms are rich in polysaccharides, proteins, and DNA, while *S. aureus* biofilms involve surface adhesins and matrix components—as demonstrated in vitro on orthopaedic implant materials [12,13,14,15,16].

*Staphylococcus epidermidis* ATCC 35984 (RP62A) and *Staphylococcus aureus* ATCC 49230 (UAMS-1) are widely used as established models for studying medical device-related infections [12,17]. RP62A is associated with chronic, low-grade implant infections, whereas UAMS-1 represents acute forms. Although UAMS-1 contributes to direct causes of PJIs and acute reactions [15], RP62A produces stable biofilm adherent to artificial implant surfaces [13,17], forming interfaces distinct from UAMS-1 biofilms, which preferentially develop on bone and soft tissue [16,17,18].

Orthopaedic implants are commonly fabricated from high-performance metallic alloys—most notably cobalt–chromium (CoCr) and titanium (Ti)—as well as from durable polymeric materials such as ultra-high-molecular-weight polyethylene (UHMWPE) [19,20,21,22]. These materials are favored for their biocompatibility, mechanical strength, and corrosion resistance and are integral to joint prostheses, spinal implants, and fixation devices [21,22,23]. Titanium alloys (e.g., TiAl_6_V_4_) are widely used in hip stems, spinal cages, and intramedullary nails due to their excellent osseointegration [21,24,25]. Cobalt–chromium alloys, known for high wear resistance, are employed in femoral components and endoprostheses for hip and knee arthroplasty [21,26,27]. In parallel, UHMWPE plays a critical role as a low-friction, wear-resistant bearing material in articulating joint replacements—including acetabular liners and tibial inserts—where it interfaces with metallic or ceramic counterfaces to facilitate smooth joint motion and minimize wear debris generation [28,29].

It is known that the physicochemical properties of biomaterials can influence bacterial adhesion and biofilm development, with certain materials promoting and others inhibiting microbial colonization [21,30,31,32,33]. Building on this premise, the objectives of this study were to quantify biofilm formation by the reference strains RP62A and UAMS-1 on orthopaedic material surfaces (discs) composed of CoCr_28_Mo_6_, TiAl_6_V_4_, and UHMWPE and to investigate in vitro biofilm formation on retrieved orthopaedic explant surfaces. In the present study, we used these well-established reference strains as experimental models, which represent chronic low-grade and acute forms of infection, respectively. Our aim was to identify potential sites of bacterial attachment and provide a representative overview of colonization patterns on commonly used implant substrates, thereby contributing to a better understanding of biofilm formation and informing strategies to prevent and control periprosthetic joint infections.

## 2. Results

### 2.1. Growth Profiles of Staphylococcus epidermidis RP62A and Staphylococcus aureus UAMS-1

**Figure 1:**
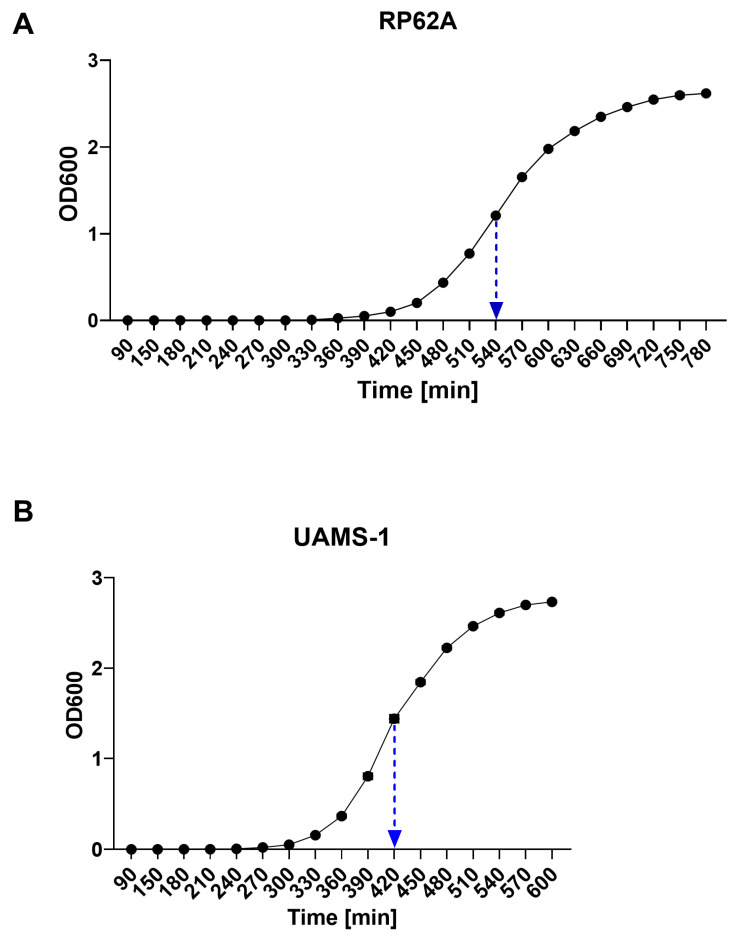
**Kinetics of bacterial growth of *Staphylococcus epidermidis* RP62A** (**A**) **and *Staphylococcus aureus* UAMS-1** (**B**). Growth was monitored by measuring OD_600_ every 30 min. Measurements were performed in three technical replicates per culture, with each experiment repeated independently twice, resulting in a total of n = 6 per time point. Data are presented as mean ± SEM.

RP62A exhibited a slower growth rate compared with UAMS-1, with an estimated log-phase doubling time of ~420 min, which was determined from the linear segment of the growth curve around 540 min (Figure 1A,B).

### 2.2. Biofilm Production by Staphylococcus epidermidis RP62A and Staphylococcus aureus UAMS-1 on CoCr, Ti and PE

We quantified biofilm formation by RP62A and UAMS-1 on cobalt-chromium (CoCr), titanium (Ti) and polyethylene (PE) discs after 72 h of incubation. All surfaces were polished to achieve nearly identical roughness, allowing us to assess the effects of material composition on biofilm formation independently of surface topography.

**Figure 2:**
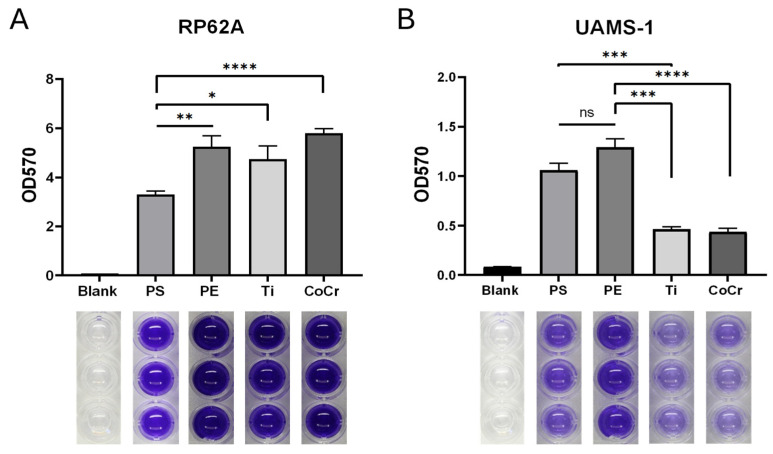
**Quantification of *Staphylococcus epidermidis* RP62A and *Staphylococcus aureus* UAMS-1 biofilm formation on disc surfaces.** Biofilm formation by (**A**) *Staphylococcus epidermidis* RP62A and (**B**) *Staphylococcus aureus* UAMS-1 on various surfaces—polystyrene (PS), ultra-high-molecular-weight polyethylene (PE), TiAl_6_V_4_ (Ti), and CoCr_28_Mo_6_ (CoCr)—was quantified using crystal violet (CV) staining. Absorbance was measured at 570 nm in three technical replicates per well, and each experiment was repeated independently twice, resulting in a total of n = 6 per surface; blank wells were also measured (n = 12). Bar graphs show mean OD_570_ ± SEM. The purple wells shown below the graphs represent the visual outcome of CV staining after solubilization with acetic acid and correspond to the OD intensity.

RP62A produced robust biofilms on all tested surfaces, with no significant differences in biofilm formation among the orthopaedic-relevant materials (CoCr, Ti, and PE). In contrast, UAMS-1 formed substantially less biofilm than RP62A, with the highest OD_570_ values observed for RP62A (5.80 ± 0.19) compared to UAMS-1 (1.29 ± 0.09) (Figure 2). Polystyrene (PS), which constitutes the bottom of the plate, was a control group and showed the lowest biofilm production for RP62A (OD_570_ = 3.30 ± 0.15). Biofilm formation on PE was significantly higher than on PS (*p* < 0.01; 60%), as was biofilm on Ti (*p* < 0.05; 44%). Notably, biofilm formation on CoCr was 76% higher compared to PS (*p* < 0.0001) (Figure 2A). Although quantitative analysis showed the highest absorbance for RP62A biofilm on CoCr, the differences compared with Ti and PE were not statistically significant (*p* > 0.1).

For UAMS-1, CoCr and Ti exhibited the lowest levels of biofilm formation, with CoCr showing the numerically lowest value (OD_570_ = 0.44 ± 0.04). Biofilm formation on PE was significantly higher compared to Ti (*p* < 0.001; 177%) and CoCr (*p* < 0.0001; 196%), while no significant difference was detected between CoCr and Ti (*p* > 0.40) (Figure 2B).

### 2.3. Visualization of Fixed Biofilms Propagated on Glass Slides

**Figure 3:**
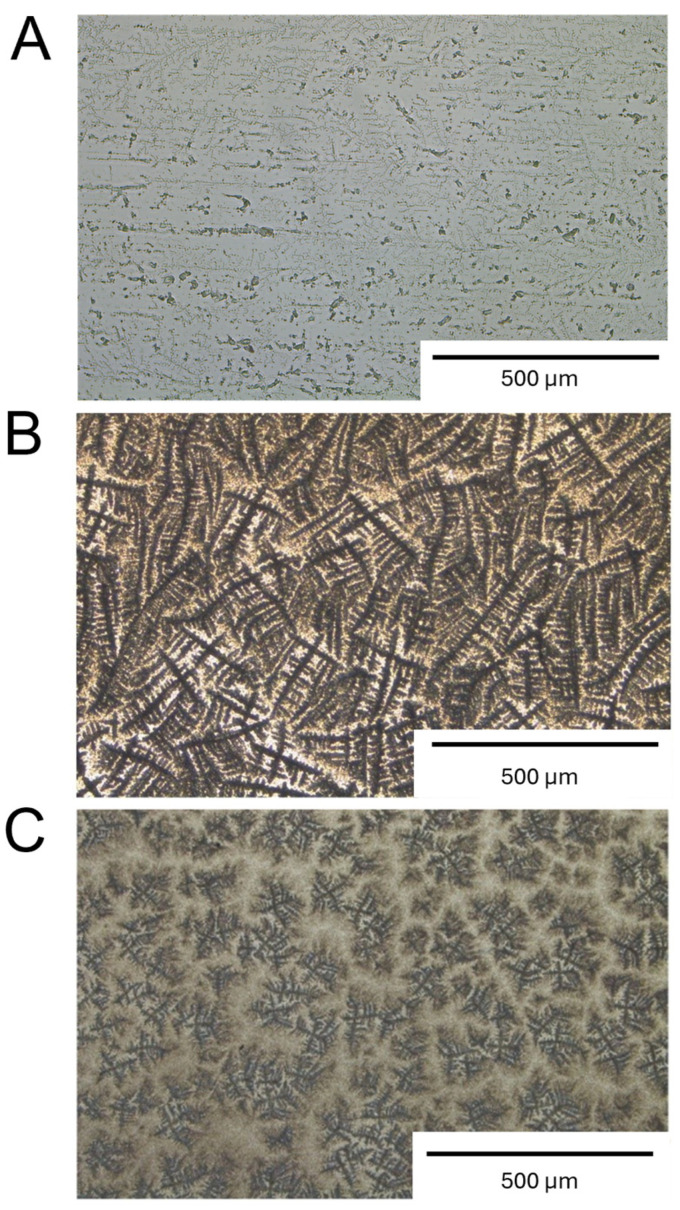
**Visualization of strain-specific surface microstructural patterns related to biofilm organization in *Staphylococcus epidermidis* RP62A and *Staphylococcus aureus* UAMS-1.** All glass slides were incubated for 72 h under the same experimental conditions. Slides were fixed with 2% formaldehyde prior to imaging. (**A**) Negative control: a slide containing only Tryptic Soy Broth (TSB) without bacteria. (**B**) *S. epidermidis* RP62A and (**C**) *S. aureus* UAMS-1. Approximately 10 images (n = 10) per slide were captured across different regions. Three slides per bacterial strain were analyzed, and a representative image is shown for each condition. Images were acquired using a LEICA brightfield microscope with a 4×/0.10 objective.

To complement the quantitative biofilm measurements obtained from material discs, glass slides were used as a visualization model to examine strain-specific surface microstructural patterns that may be related to biofilm organization. Biofilms of *Staphylococcus epidermidis* RP62A and *Staphylococcus aureus* UAMS-1 cultured for 72 h displayed distinct dendritic, branch-like geometries on the glass substrate (Figure 3).

Glass slides incubated in TSB alone served as the negative control (Figure 3A). RP62A (Figure 3B) showed a dense and highly interconnected network of elongated, ridge-like structures covering almost the entire field of view. The individual “branches” were long (approximately 300 µm), curved, and frequently bifurcating, forming a tightly packed, weave-like pattern. The ridges appeared darker than the surrounding background, indicating areas of higher deposited material.

In contrast, UAMS-1 (Figure 3C) exhibited a more heterogeneous and discontinuous pattern. The features were noticeably shorter (approximately 50 µm), more fragmented, and distributed, with larger gaps between clusters. The overall organization appeared less interconnected, with smaller radial aggregates and reduced continuity compared to RP62A. These observations describe surface patterns visualized at low magnification and are presented as structural features that are potentially associated with biofilm organization.

### 2.4. Surface-Dependent Biofilm Development of Staphylococcus epidermidis RP62A on Retrieved Orthopaedic Components

Since RP62A produced the most cohesive biofilms in previous assays, explant experiments were performed exclusively with this strain to evaluate biofilm formation on orthopaedic implant explants under in vitro conditions. A 72-h in vitro biofilm cultivation experiment was performed on various knee implant explants, followed by fixation and crystal violet (CV) staining for visualization. All bone-facing surfaces exhibited a rough topology, whereas joint-facing ones were polished.

**Figure 4:**
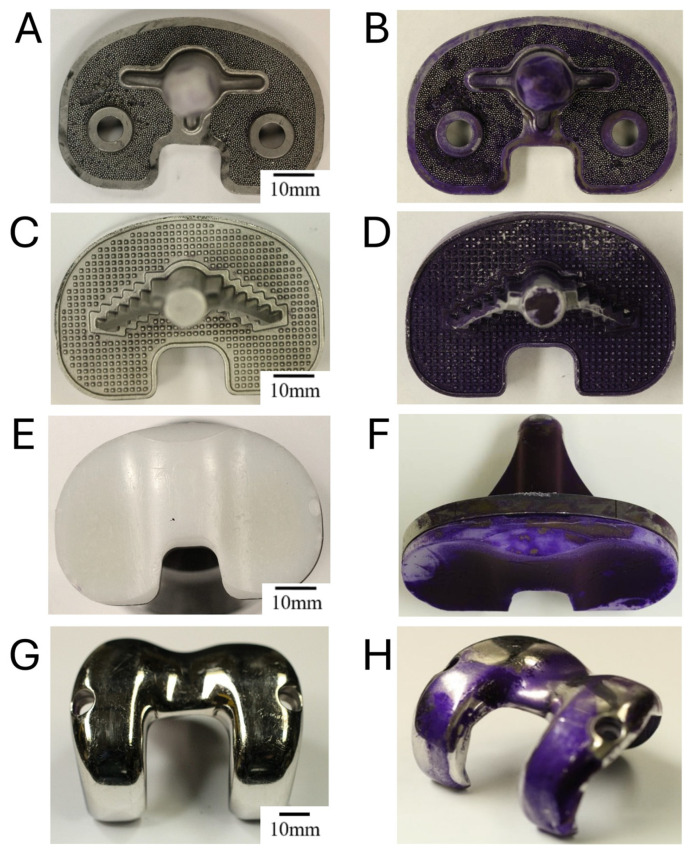
**Stained Biofilm deposits produced by *Staphylococcus epidermidis* RP62A on orthopaedic explant components of artificial knee joints.** RP62A biofilms were cultivated for 72h, fixed in formyl aldehyde and stained with crystal violet (CV) for visualization and image acquisition. Implant parts in images are TiAl_6_V_4_ tibial baseplate (**A**,**B**); TiAl_6_V_4_ Stryker tibial baseplate (**C**,**D**), PE tibial insert (**E**,**F**); CoCr polished joint-facing femoral component (**G**,**H**). (**A**,**C**,**E**,**G**) correspond to images of implant components after the dye was washed off and (**B**,**D**,**F**,**H**) represent the components after fixation and staining with CV dye.

We assessed two different tibial baseplate components with distinct topologies. The first implant featured a porous TiAl_6_V_4_ titanium alloy surface (Figure 4A,B), typically used to promote bone ingrowth or cement interdigitation.

The designed structure of the tibial baseplates is beneficial for osseointegration but also presents an increased risk of bacterial colonization and biofilm formation. The next explant component (Figure 4C,D) is a Stryker TiAl_6_V_4_ tibial baseplate used in total knee arthroplasty, featuring a central fixation stem and two ridged anchoring surfaces to enhance mechanical interlock with bone or cement. The textured grid pattern promotes cement interdigitation. Consistent CV staining was detected on rough Ti alloy tibial baseplates, suggesting biofilm biomass on the bone-facing surfaces (Figure 4B,D). These surfaces are typically microporous or ridged, which may provide surface characteristics that facilitate bacterial attachment and biofilm formation under the in vitro conditions used in this study.

The next component is a polyethylene (PE) tibial insert, which articulates with the femoral component in total knee arthroplasty (Figure 4E). It is designed to sit atop the tibial baseplate and features medial and lateral condylar surfaces that accommodate femoral motion, allowing load distribution and joint stability. The PE tibial insert (Figure 4F) showed the highest accumulation of crystal violet (CV) stain, indicating substantial biofilm formation. The more intensely stained purple areas correspond to regions of increased biofilm attachment. These regions coincide with surfaces that experience greater implant movement and articulation. In addition, stronger CV staining was observed along edges, cracks, and interfacial zones where different materials meet.

Figure 4G,H depict the cobalt–chromium (CoCr) femoral component. This component typically articulates with the polyethylene tibial insert (Figure 4E) and replicates the geometry of the femoral condyles to enable smooth flexion and extension. The CoCr femoral component (Figure 4H) exhibited minimal CV staining on its polished, joint-facing surface, indicating low levels of biofilm accumulation. The absence of intense staining was observed on these smoother surface regions.

In total, the lowest biofilm presence was seen in the polished part of the femoral component, which showed some presence of a uniform layer of CV dye (Figure 4H); however, it showed no substantial biofilm deposits in comparison to metal tibial baseplates and a plastic tibial insert (Figure 4B,D,F).

## 3. Discussion

*Staphylococcus epidermidis* and *Staphylococcus aureus* are well known for their strong biofilm-forming capacity and are among the leading causes of biomaterial-associated infections [12,13,14,15,16]. *Staphylococcus epidermidis* is recognized as an opportunistic pathogen that primarily causes infections in immunocompromised patients with implants and is frequently isolated from prosthetic joint infections (PJIs) as well as from implants removed due to aseptic prosthetic loosening [6,33,34,35]. *Staphylococcus aureus* is also a major cause of biofilm-associated infections, particularly on implanted medical devices [7,36,37].

In a corresponding study, we investigated biofilm formation by two clinically relevant bacterial strains: *S. epidermidis* (RP62A) from a catheter-associated infection and *S. aureus* (UAMS-1) from osteomyelitis [17,18]. Both strains are frequently used to test the strength of biofilm-inhibiting agents, medical device coatings, and host immune responses to biofilms [12,17,38,39]. Biofilms formed by *S. aureus* strains such as UAMS-1 can be present on surrounding tissues and bone at the implant–bone interface and have been associated with acute, aggressive prosthetic joint infections (PJIs) characterized by bone destruction, abscess formation, and inflammation [15,33,40]. RP62A has been implicated in chronic low-grade PJIs and characterized by dense, persistent biofilm formation on surfaces [16,17,34].

UAMS-1 demonstrated faster growth kinetics than RP62A, whereas RP62A reached a higher optical density during the log phase, reflecting greater culture turbidity (biomass). Differences in growth dynamics between *S. aureus* and *S. epidermidis* are well documented, with *S. aureus* typically favoring rapid planktonic replication and *S. epidermidis* exhibiting slower growth associated with a more persistent, biofilm-oriented lifestyle [34,35].

These intrinsic physiological differences justify the use of extended incubation for in vitro biofilm studies as they allow both rapid and slow-growing strains to establish mature, surface-attached biofilms. Accordingly, the 72-h incubation period applied in this study was selected to ensure the formation of stable, mature biofilms, consistent with previous in vitro models evaluating surface-dependent differences and biofilm resilience across various biomaterials [13,41,42,43,44]. Under these conditions, RP62A produced markedly more biofilm compared with UAMS-1. Notably, CoCr, Ti, and polyethylene (PE) supported RP62A biofilm formation to a similar extent as polystyrene (PS), while metallic surfaces displayed a clear inhibitory effect on UAMS-1. These results align with previous studies demonstrating that metallic substrates, particularly Ti and CoCr alloys, can reduce *S. aureus* adhesion and biofilm formation compared to polymeric materials such as PE [28,45]. In addition, a recently conducted investigation also reported broad-spectrum antibacterial activity across Ti and CoCr implant materials, consistent with our observations [32,46,47].

Consistent with the controlled in vitro observations, experiments performed using orthopaedic implant explants showed similar patterns of biofilm distribution across the tested materials. In this model, biofilms of RP62A grown on the explant surfaces were more pronounced on polyethylene (PE) components—particularly the tibial insert—than on the metal alloy surfaces. This observation is consistent with another study, which revealed larger bacterial counts after sonication of PE liners than of metal alloys [48]. These regions of the PE tibial component undergo repeated movement during joint function, leading to higher frictional stress and microscopic surface abrasion. The resulting roughened microtopography facilitates bacterial adhesion and subsequent biofilm maturation [49,50,51]. Such wear-induced irregularities are also associated with localized tribocorrosion, where mechanical and biochemical factors interact to promote both material degradation and microbial colonization [13,52,53]. In addition, stronger CV staining was observed along the edges, cracks, and interfacial zones where different materials meet, suggesting that these transition areas provide sheltered microenvironments that favor bacterial attachment and biofilm accumulation. Based on our findings, it appears that surface properties—such as hydrophobicity, micro-/nanotopography, and elemental composition—may exert a more pronounced influence on *S. aureus* biofilm formation than on that of *S. epidermidis*. This could be explained by the stronger dependence of *S. aureus* on surface-mediated adhesion mechanisms, while *S. epidermidis* forms biofilms more uniformly through polysaccharide intracellular adhesion (PIA), regardless of substrate type [54,55]. This observation holds particular relevance for orthopaedic implants, where targeted surface engineering strategies could be leveraged to modulate microbial colonization and reduce the risk of device-associated infections.

Given that implant components of a total knee arthroplasty system can act as substrates for biofilm growth [4,48,56], their capacity to support biofilm formation was assessed. The tibial baseplates composed of titanium alloy displayed prominent biofilm production, which might be explained by the fact that these surfaces also create microenvironments that support bacterial adherence and biofilm maturation [57,58,59], which readily colonize rough surfaces through polysaccharide-mediated adhesion [55].

In contrast, the PE tibial insert and cobalt-chromium alloy femoral component contain smoother surfaces, which are usually known to be less supportive for initial bacterial adhesion and biofilm formation [32,45,47]. However, they can still harbor biofilms when proteinaceous conditioning films (e.g., fibrin, plasma proteins) are present [33,47]. The observed higher biofilm adhesion on PE surfaces in the present study may be attributed to the unmodified nature of PE, as polyethylene inserts are typically not subjected to surface treatments such as roughening or antimicrobial coatings that are commonly applied to metallic implant components [31,32,33,46]. From a clinical perspective, similar or even more pronounced effects may be expected, as retrieved polyethylene components are exposed to material aging, oxidative degradation, and wear-related surface alterations within biological environments. These processes may further promote microbial adhesion and biofilm persistence, thereby increasing the risk of implant-associated infections and complicating eradication strategies during revision procedures.

In our study, *S. epidermidis* RP62A appeared to heavily colonize the PE tibial insert, forming a dense biofilm layer. Although quantitative assessment was not performed and evaluation was based on visual inspection, the biofilm on the PE insert appeared notably thicker than on metal alloy components. This contrasts slightly with our disc experiments, which showed similarly high biofilm formation on both polished PE and metal surfaces. This PE surface of the tibial insert was modified (degraded) by coming into contact with the biochemical environment inside the human knee, producing small changes in topology, which favored biofilm production by RP62A. In addition, the PE explant component was not polished in comparison to tested disc surfaces. This might have influenced biofilm formation patterns.

RP62A demonstrated tenacious biofilm formation on abiotic surfaces, particularly those relevant to orthopaedics, which is consistent with many other studies [13,14,17,39,60]. In contrast, under the experimental conditions of this study, UAMS-1 exhibited lower levels of biofilm formation on all tested implant material discs compared with RP62A. However, our model is limited to interaction of artificial materials with bacterial biofilms, which does not include biological tissues. Since UAMS-1 is more associated with PJIs characterized by biofilm formation on bones and tissues [12,18], it explains its inability to produce biofilm on artificial materials. Future studies within our group are planned to address this gap by examining biofilm formation on periprosthetic substrates such as bone, cartilage, and synovial-derived materials, which more closely recapitulate the native joint microenvironment [57,58].

As with any in vitro study, several limitations must be acknowledged that may impact the interpretation and translational relevance of the findings. A key limitation is the relatively short duration of biofilm cultivation. While RP62A and UAMS-1 biofilms were grown for 72 h, extending the incubation period to several weeks could better capture the long-term development and maturation of in vitro biofilms. It is plausible that the structure and composition of long-term biofilms differ significantly from those of early-stage biofilms [61,62]. Moreover, prolonged incubation may reveal changes in biofilm stability and adhesion, potentially leading to material-dependent variations in biofilm integrity over time.

Currently, there is very limited information on RP62A and UAMS-1 interactions [63]. Future studies should also investigate the interactions between these two strains in polymicrobial communities. Examining how these strains engage in symbiotic or competitive relationships during co-colonization could provide valuable insight into their roles in pathogenesis and biofilm persistence in vivo. In addition, prior studies have explored the effects of antibiotic-loaded coatings on metal implants in limiting *S. aureus* biofilm formation [38], which represents another relevant and interesting direction for future investigation in the context of the present work.

## 4. Materials and Methods

### 4.1. Kinetics of Biofilm-Producing Bacterial Strains

The microorganisms used in our experiments were well-established reference collection strains obtained from recognized culture collections. The bacterial collection strains were: *Staphylococcus epidermidis* ATCC 35984 (RP62A), originally isolated from a case of catheter sepsis [64], and *Staphylococcus aureus* ATCC 49230 (UAMS-1), originally isolated from a patient with chronic osteomyelitis at the McClellan Veterans Hospital in Little Rock [65]. Both strains were stored at −80 °C using a cryopreservation system (Microbank™, Pro-lab Diagnostics, Toronto, ON, Canada). Liquid cultures were prepared for each bacterial strain from cryogenically stored Microbank™ beads. A single bead, corresponding to approximately 2 × 10^5^ colony forming units (CFU)/mL, was inoculated into 100 mL of Tryptic Soy Broth (TSB) (Merck Millipore, Darmstadt, Germany) in 200 mL flasks and incubated at 37 °C with shaking at 150 rpm. Each growth experiment was performed in two independent biological repeats, with three technical replicates per strain. Growth kinetics were monitored by measuring the optical density at 600 nm (OD_600_) in 1 mL cuvettes (Plastibrand, dimensions of 12.5 × 12.5 × 45 mm, 1.5 mL semi-micro cuvettes, Cat. No. 7590 15, BRAND GmbH + Co. KG, Wertheim, Germany) using a photometer (Eppendorf AG, Hamburg, Germany) every 30 min until a decline in growth rate was observed. Growth curves were generated using GraphPad Prism 10.6.1.

### 4.2. Discs and Surface Modification

The test specimens were fabricated from materials commonly used in orthopaedic implant production. All specimens were manufactured by a certified medical device company in accordance with DIN EN ISO 13485. Surface treatments were performed according to the specifications of a typical hip implant designed for cement-free fixation. Discs were produced from three materials—cobalt–chromium alloy (CoCr_28_Mo_6_; CoCr), titanium alloy (TiAl_6_V_4_; Ti), and ultra-high-molecular-weight polyethylene (UHMWPE; PE)—each machined into discs measuring 10 mm in diameter and 3 mm in thickness. To ensure uniform surface smoothness, all samples were processed using a QuickTech Ultimate I60 turning–milling center equipped with a Mitsubishi control system, with machining parameters optimized for each material (Quick-TECH Machinery Co., Ltd., Kaohsiung City, Taiwan). CoCr discs were machined at 2070 rpm with a feed rate of 0.07 mm/rev using coolant-assisted machining (6% Blaser Synergy 735). Ti discs were machined at 2500 rpm with the same feed rate and coolant conditions. PE discs were dry-machined at 4000 rpm with a feed rate of 0.15 mm/rev. After machining, all discs were cleaned with 5% Olschner 9.1 SP detergent, rinsed thoroughly with deionized water in a Miele Professional G7863 CD laboratory-grade washer, air-dried under sterile conditions, and stored at room temperature (RT, 23 ± 1 °C) until subsequent biofilm experiments.

### 4.3. Biofilm Cultivation and Quantification with Crystal Violet

Biofilm formation by *S. epidermidis* (RP62A) and *S. aureus* (UAMS-1) was assessed on clinically relevant orthopaedic material/disc surfaces and control substrates. The biofilm quantification protocol using crystal violet (CV) staining was adapted and modified from Christensen et al., 1985 [66]. Bacterial cultures were prepared by inoculating four colonies from agar plates into 30 mL of Tryptic Soy Broth (TSB) and incubating them at 37 °C with shaking (150 rpm) for approximately 5 h, corresponding to late exponential/early stationary growth phase. Optical density at 600 nm (OD_600_) was measured using a photometer, and cultures were diluted in fresh TSB to an OD_600_ of 0.008 ± 0.005, corresponding to approximately 1.5 × 10^6^ colony-forming units (CFU) per well, as determined by parallel CFU quantification. This number of bacteria has been found to be optimal for incubation periods of 72 h. Viable cell counts were validated using a spot plating assay. Briefly, aliquots were taken from diluted cultures, subjected to seven serial ten-fold dilutions, and 10 µL of each dilution was plated onto divided tryptic soy agar plates. Colonies were counted after 20 h of incubation at 37 °C, and CFU/mL was calculated as (number of colonies × dilution factor)/plated volume. Orthopaedic material discs were autoclaved, mounted in sterile 24-well tissue culture plates using 3 µL of cyanoacrylate adhesive, and dried overnight. Microscopic examination confirmed that autoclaving did not alter the polished surfaces of the metallic and non-metallic discs. Each disc was inoculated with 1mL of the diluted bacterial suspension and incubated at 37 °C under static conditions for 72 h. Each experiment was performed in two independent biological repeats, with three technical replicates per bacterial strain and surface type. Each well received 1 mL for wells with discs (24-well plates, Costar, Corning, NY, USA), ensuring full coverage of the implant surface and uniform bacteria–substrate contact, and 0.5 mL for wells without discs (48-well plates, Falcon, Corning, NY, USA; the well diameter approximates disc size to avoid excess liquid), and the growth medium (TSB) was replaced every 24 h to maintain nutrient availability; this setup also accounts for gravity-driven biofilm formation on horizontal surfaces, ensuring consistent growth across replicates. After incubation, wells were gently rinsed with Dulbecco’s phosphate-buffered saline (DPBS) to remove planktonic bacteria and checked for contamination on Tryptic Soy Agar. Biofilms were fixed with 2% formaldehyde for 5 min, dried for 30 min and stained with 0.13% CV; Carl Roth GmbH + Co. KG, Karlsruhe, Germany for 10 min, rinsed thoroughly, and dried at 50 °C. Bound dye was solubilized with 30% acetic acid, and absorbance was measured at 570 nm using a TECAN microplate reader. Biofilm biomass was normalized by subtracting the mean CV absorbance of blank wells for each surface type. The assay’s linear range was verified using a standard curve of CV dilution, showing linearity between approximately 0.1 and 3.5 absorbance units (R^2^ = 0.999), indicating that the measured OD values fall within the reliable detection range of the plate reader.

### 4.4. Biofilm Visualization on Glass Slides

The glass slides (MirrIR Low-E, Kevley Technologies, Chesterland, OH, USA) were used as reference surfaces to enable comparative visualization of biofilm coverage patterns under controlled and optically uniform conditions, independent of the surface roughness and opacity of metallic implant materials. This approach allowed for consistent microscopic observation of the spatial distribution of bacterial deposition while maintaining surface chemistry relevance [67]. Additionally, the use of these slides enabled visualization of biofilm coverage patterns independent of surface roughness, curvature, and opacity characteristic of metallic implant materials while preserving surface chemistry effects. Slides were sterilized by immersion in 100% ethanol for 30 min, followed by ultraviolet (UV) exposure for 15 min on each side. Overnight bacterial cultures were diluted in Tryptic Soy Broth (TSB) to the same inoculum density used in disc experiments (OD_600_ = 0.008 ± 0.005, corresponding to approximately 1.5 × 10^6^ CFU/mL) and applied to the prepared slides in a final volume of 25 mL per 100 mm Petri dish (Cat. No. C-8213; NeoLab GmbH, Heidelberg, Germany). Biofilms were cultivated for 72 h at 37 °C under static conditions, with daily medium replacements. Following incubation, slides were gently washed with sterile phosphate-buffered saline (PBS) to remove non-adherent bacteria, fixed with 2% formaldehyde for 5 min, and air-dried prior to visualization. The slides were imaged using brightfield microscopy with a 4×/0.10 objective.

### 4.5. Biofilm Cultivation on Retrieved Orthopaedic Implant Components

Retrieved prosthetic components (knee implants) from patients included in this study were analyzed retrospectively in a fully anonymized manner. Furthermore, the materials used in this study originated from a certified arthroplasty center and were handled in accordance with established institutional procedures. All material components were archived exclusively in anonymized form without any possibility of patient identification. The implants were sterilized by autoclaving and subsequently used as substrates in an in vitro model to specifically investigate biofilm formation on explanted, worn surfaces. This experimental step was performed to qualitatively assess biofilm formation on the actual worn regions of different implant materials and to visualize material-specific differences for descriptive purposes. The implants included tibial baseplates, polyethylene (PE) inserts, and femoral components composed of cobalt–chromium (CoCr_28_Mo_6_) and titanium (TiAl_6_V_4_) alloys. To facilitate biofilm formation, the worn explant surfaces were exposed to freshly prepared *Staphylococcus epidermidis* RP62A cultures diluted to an optical density (OD_600_) of 0.008 ± 0.005, corresponding to approximately 1.5 × 10^6^ CFU/mL, in 500 mL. The samples were incubated in vitro under static conditions at 37 °C for 72 h, with the growth medium (Tryptic Soy Broth, TSB) replaced every 24 h to maintain nutrient availability. Following incubation, each component was rinsed with DPBS to remove non-adherent bacteria, fixed for 10 min in 4% formaldehyde (FA), and dried at 50 °C for approximately 90 min. Explants were then stained with 0.13% crystal violet (CV), dried again for 120 min at 50 °C, and photographed using a Canon EOS 7D digital camera under standardized lighting conditions (Canon Inc., Tokyo, Japan).

### 4.6. Statistical Analysis

Statistical analyses were performed using GraphPad Prism 10.6.1 (GraphPad Software, Inc., Boston, MA, USA). Data are presented as mean ± SEM unless otherwise stated. Differences in biofilm formation between material groups were assessed using Welch’s *t*-test, which does not assume equal variances between samples, with *p* < 0.05 considered significant. All tests were two-tailed. Significance levels are reported as: *p* ≤ 0.05 (*), *p* ≤ 0.01 (**), *p* ≤ 0.001 (***), and *p* ≤ 0.0001 (****).

## 5. Conclusions

This study provides a comprehensive in vitro assessment of biofilm formation by *Staphylococcus epidermidis* (RP62A) and *Staphylococcus aureus* (UAMS-1) on orthopaedic materials/disc surfaces, including cobalt–chromium (CoCr), titanium (Ti), and polyethylene (PE), as well as on retrieved explant components. RP62A formed consistently dense biofilms across all substrates, whereas UAMS-1 produced substantially less biofilm on metallic surfaces, demonstrating strain- and material-dependent differences in biofilm formation. Observations on glass slides were performed to examine strain-specific surface microstructural patterns that may be related to biofilm organization. These findings highlight variability in biofilm architecture and distribution in vitro and provide a basis for further studies using extended cultivation models and polymicrobial systems to explore biofilm behavior under more complex laboratory conditions.

## Data Availability

Datasets are available on request. The raw data supporting the conclusions of this article will be made available by the authors without reservation.

## Abbreviations

°C	degrees Celsius
CFU	colony-forming units
CoCr	cobalt-chromium alloy CoCr_28_Mo_6_
CV	crystal violet
DNA	deoxyribonucleic acid
DPBS	Dulbecco’s phosphate-buffered saline
e.g.,	exempli gratia
h	hour
µL	microlitre
min	minute
mL	millilitre
mm	millimetre
mm/rev	millimeters per revolution
nm	nanometre
OD	optical density
PBS	phosphate-buffered saline
PE	polyethylene
PIA	polysaccharide intracellular adhesion
PJI	periprosthetic joint infection
PS	polystyrene
Ref.	reference
RP62A	*Staphylococcus epidermidis* ATCC 35984
rpm	rounds per minute
SEM	standard error of the mean
Ti	Titanium alloy TiAl_6_V_4_
TSB	Tryptic Soy Broth
UAMS-1	*Staphylococcus aureus* ATCC 49230
UHMWPE	ultra-high-molecular-weight polyethylene
USA	United States of America
UV	ultraviolet
